# You Are What You Eat… But Do You Eat What You Are? The Role of Identity in Eating Behaviors—A Scoping Review

**DOI:** 10.3390/nu14173456

**Published:** 2022-08-23

**Authors:** Suzannah Gerber, Sara C. Folta

**Affiliations:** 1 Gerald J. and Dorothy R. Friedman School of Nutrition Science and Policy, Tufts University, Boston, MA 02111, USA; 2 Betty and Guy Beatty Liver and Obesity Research Program, Inova Medical System, Falls Church, VA 22043, USA

**Keywords:** diet, identity, self-concept, psychology, culture, behavior, food, acculturation

## Abstract

Background: Identity is a major construct in the fields of psychology and anthropology that can relate to both the maintenance of eating behaviors and cultural sensitivity. However, there has not been any systematic effort to understand the role of identity in eating behaviors and the maintenance of eating behaviors, or to address multiple aspects of identity within an individual across scientific disciplines. This scoping review aims to understand and describe existing research relating identity to eating behaviors and to detail the measurement of identity. Methods: We conducted a systematic search of Ovid, PsychINFO, Embase, and Web of Science for articles on identity and eating behaviors published between January 1946 and March 2022. We utilized the Preferred Reporting Items for Systematic reviews and Meta-Analyses extension for Scoping Reviews (PRISMA-ScR) checklist, and search methods were developed with the assistance of a research librarian. We rated articles from 1 to 5 based on the depth, complexity, and multi-dimensionality of the identity measurement conducted. Scoring criteria included a review of the number of items directly querying or evaluating identity and the extent of measurement of identity domains. Results: In total, 100 articles were included, examining 10 different identities, 8 identity constructs, 11 eating behaviors, and construct contributions from 26 theories. The mean score of all articles was 2.9 on the scale from 1 to 5. A total of 10 studies scored a “1”; 30 scored a “2”, indicating the use of 1–2 basic questions about identity; 31 received a “3” for use of a common but non-complex identity instrument; 19 received a “4”, meaning they contained strong evaluation and included multiple types of identity but were lacking in terms of depth of measure and/or the comparison of identity effects to constructs; and 10 scored a “5” for their strong, in-depth measure of identity and inclusion of multiple types. Identity was found to be significantly related to eating behaviors in all but one study. Conclusion: Identity measurements seldom accounted for complexities such as multiple identities and identity shifting over time. Nonetheless, our findings indicate that multiple aspects of identity reciprocally reinforce behavior and that change maintenance is associated with identity salience and centrality. Identity is underutilized and heterogeneously applied in eating behavior research. The inclusion of identity assessments may lead to better outcomes being obtained within differing cultural, normative, and environmental scenarios.

## 1. Introduction

Poor diet is the leading cause of non-communicable disease globally [1,2]. It is responsible for approximately 11 million deaths and 255 million disability-adjusted life years (DALYs) annually, a number which is growing [1]. There is scientific consensus regarding the importance of diet in the prevention and treatment of chronic illness [1,2,3,4,5]. However, most interventions designed to change dietary behavior are of short duration and may be ineffective in achieving long-term change in individuals [6]. Therefore, innovative approaches to long-term dietary behavior change are urgently needed. Given the global nature of this issue, the social disparities that exist in diet quality [7,8,9,10], and the broad role of identity in health behaviors [11,12], it is especially important to identify strategies that can fully account for personal, social, and cultural factors.

### 1.1. Multi-Disciplinary Theories of Identity

Identity is a major construct in the fields of psychology and anthropology that may be relevant to eating behaviors. While many theories of identity exist within these disciplines, the most commonly applied within the eating behavior literature stem from two main theories: Identity Theory (IT) and Social Identity Theory (SIT). According to IT, identities are evolving and multifold [13,14,15]. For instance, an individual may be simultaneously a mother, a software developer, a vegetarian, and a recent immigrant, and each of these identities will have a range of internal motivations, values, and goals. IT aims to assess how an individual’s multiple identities interrelate and how they may vary in centrality and in salience by context [13,16,17,18,19,20]. For example, individuals may shift among components of their identity, thinking and acting somewhat differently depending on whether they are at work or with family. Identities may also shift and change in response to more profound changes in context, such as developing a major illness or immigrating to a new country.

Social Identity Theory (SIT), a second major psychological theory of identity, aims to understand identity as a social construct that develops from group membership and social affiliations [21]. SIT examines the role that identification with a group (in-grouping) plays in the reinforcement of behavior, as well as the anticipation, or fear, of judgement for failing to behave congruently with the group. SIT examines the motives and pressures of conformity, such as in-group stigma and out-group discrimination, and the cognitive dissonance of identity threat when individual members violate their own beliefs [22]. In both IT and SIT, behavior reinforces one’s sense of self—values, social roles, and normative expectations—and signals affinity to groups with whom an individual identifies.

In the realm of health behavior theory, there has been increasing interest in including identity [23,24,25], most notably in the Theory of Planned Behavior (TPB). Self-identity was first introduced in the TPB to help account for the salience of internal values as a predictor of intention [26,27]. As such, it is thought to help understand and predict differences in the intentions to perform and sustain behavior based on how important the behavior is to the way someone describes themselves [26,27,28,29]. Self-identity is one of the most common extensions of the TPB and has been shown in multiple meta-analyses to explain an additional 4–6% of the variance in intention and up to 9% when controlling for past behavior [28,30,31,32,33,34].

### 1.2. Prior Understanding of Identity and Eating Behaviors

In anthropology, food is considered a central part of identity formation [35,36,37]. Using food, cultures demonstrate in-group affiliation, within-group hierarchies, and within- and between-group rituals that reinforce cultural identity [35,38]. How food is chosen, made, served, and eaten all serve to co-construct identities of multiple orders—self-identity, family identity, ethnic identity, national identity, religious identity, and others [39,40,41,42]. On an individual level, the relationship between food and identity is thought to be even more connected. Food choices signal active allegiances with social groups and reinforce norms, stereotypes, and beliefs; and they do so multiple times a day, throughout an individual’s lifespan [35,37,43]. However, the complex and independent effect of identity on eating behaviors has not been extensively summarized.

Changes in eating behaviors require individuals to make effort to change their habits, maintain the change, and perform continual decision-making relevant to that change. It therefore seems reasonable to suggest that identity processing and salience may help explain variations in both the initiation and maintenance of eating behaviors. However, there is scant literature that actively investigates how to capture various identities and how their salience shifts and evolves with respect to eating behaviors [25,35,44,45,46,47,48,49]. Additionally, the roles of identity, identity formation, contextual identity shifting, and identity change throughout the lifespan are also not a major domain of the health behavior theories that are most frequently used to guide changes in eating behaviors. Indeed, identity is only minimally discussed in the major textbooks widely used to train professionals in health behavior theory [50].

Nine known reviews look at the relationship between eating behaviors and identity [28,36,37,38,43,51,52,53,54]. The most recent review published in 2019 summarized the role of identity in the health behavior of retired athletes [52]. The next most recent in 2018 attempted to describe weight stigma and identification as an overweight person with weight loss attempts [53], and the third most recent in 2016 summarized multiple barriers to health behavior adoption in mid-life women [54]. Other reviews looked at the role of identity more directly, the most recent of which was focused on social influences and was published in 2013 [43]. Most reviews focus on singular types of identity (e.g., self-identity), and/or limit themselves to specific food choices (such as organic food) rather than examining the effects of simultaneous multiple identities or more complex eating behaviors and patterns. Furthermore, most reviews look at the relationship of identity with “healthy eating behavior”—broadly defined—despite a rich literature base exploring other food choices, social eating, ethnic eating behavior, ethical eating behavior, disordered eating, and other dietary patterns. A new review of the topic that compares different identity types and different methods for measuring those identities is warranted.

### 1.3. Aims

The aims of this review are to (1) describe the operationalization of identity within the eating behavior literature and (2) summarize the current evidence on the role of identity in eating behaviors, drawing from the literature across different disciplines and methodologies.

## 2. Methods

A scoping review was undertaken, since the literature on this topic spans multiple disciplines, uses both qualitative and quantitative approaches, and employs multiple types of analytical methods. A scoping review is a systematic review that allows for mixed-methods synthesis and summary and adheres to the PRISMA guidelines for systematic reviews of this type (PRISMA-ScR) [55]. We were interested in including all relevant works on the topic, including older works if discoverable, given that foundational work on identity emerged in the mid-20th century. A search was performed on articles published between January 1946 (the earliest setting in the databases) and 30 March 2022, on Ovid, PsychInfo, and Embase using combinations of keywords and database-specific controlled vocabulary in titles, abstracts, and indexing fields. We utilized the Preferred Reporting Items for Systematic reviews and Meta-Analyses extension for Scoping Reviews (PRISMA-ScR) Checklist [55], and search methods were developed with the assistance of a research librarian. Search terms used can be found in the full search example provided in the Supplementary Material (Search Strategy for OVID in Electronic Supplemental Material 1; PRISMA-ScR Checklist in Supplemental Material 2). To ensure no relevant literature was overlooked, articles were gathered from the works cited and from articles citing chosen articles using Web of Science with the same inclusion/exclusion criteria applied.

Articles were included if they: (1) focused on eating behaviors and/or dietary change, or the intent to change diet; (2) studied humans; (3) included a direct measure of “identity” (including “self-concept” and “self-schema”); (4) reported on intervention studies, observational studies, and systematic reviews specific to eating/diet and identity; and (5) were published in peer-reviewed journals. Identity based on sociodemographic characteristics was only included if the study directly measured the strength of the subjective importance, affiliation, or level of self-identification with that sociodemographic identity. Both authors reviewed articles and came to a consensus regarding article inclusion.

Articles were excluded if: (1) identity was not specifically measured, was not treated as a dependent or independent variable in assessments, or was only used to describe the sample in sociodemographic terms; (2) they solely examined beverage consumption or physical activity without eating behaviors; (3) they were funded by a commercial funder with a potential conflict of interest (such as in the food and beverage industry); (4) they described the development of survey instruments; (5) the eating behaviors were not the voluntary behaviors of the participant, such as parent feeding behavior, clinical care practice or attitudes, or from teaching curricula; (6) behaviors were temporary, involuntary, or not relevant to lifestyle maintenance, such as pregnancy diets, parenteral feeding, or the eating behaviors of the institutionalized; (7) no full text was available or was not available in English; or (8) they described assessments of risk behavior (e.g., unhealthy eating clustered with other risk behaviors such as disordered eating, substance use, smoking, and sexual behaviors).

### 2.1. Data Charting

Data extraction was performed by the first author using a modified protocol template from JBI Reviewer’s Manual [56] in conjunction with the PRISMA-Sc checklist [55] (PRISMA-ScR Checklist in Supplemental Material 2). The template was expanded to include domains relevant to the study: (1) eating behavior type; (2) population/sociodemographics; (3) duration of study; (4) type of identity; and (5) the identity theory used, if any. Both authors reviewed and found consensus for article sorting by type and strength when evaluated.

### 2.2. Scoring of Identity Measurement

We constructed a scoring system to provide a better understanding of the measures used and to help describe their adequacy. We rated articles based on the depth, complexity, and multi-dimensionality of the identity measurement in the study on a scale from 1 to 5. Scoring criteria were inductively derived based on the identity theories most commonly used in the literature on this subject. To score the measures, we reviewed the number of items directly querying or evaluating identity and the inclusion and extent of the measurement of identity domains, as follows:

Score of 1: Little direct measurement of identity. Little to no detailed reporting of identity in results but discussion indicates the significance of identity to participants or to study findings, or if it is a topic offered by a subject in a qualitative setting rather than a measure derived from a study instrument.

Score of 2: Uses basic questions about identity (e.g., “I identify as a healthy eater”), and only 1 or 2 such questions, with little to no emphasis on the centrality or magnitude of that identity. Typically, identity is assessed as a prompted declaration, with no internal or reflective evaluations.

Score of 3: Uses a common identity instrument (such as self-identity instrument in TPB) consisting of 3–5 items but does not account for more complex components such as change, formation, multiple identities, or reasons for change in identity or behaviors. Self-evaluation and reflection may be present but are limited and focused on behavior as evidence of the identity, or identity as the effect modification for the behavior or intention.

Score of 4: Strong evaluation of identity and multiple types of identity, but may lack depth in considering identity—for example, in the pre-cognitive aspects or identity process; may not compare identity effects to other constructs evaluated in the same study (e.g., interaction with self-efficacy or independence from norms). These studies may also use additional instruments to help explain identity (such as self-perception).

Score of 5: Strong and in-depth evaluation of identity, including multiple types of identity. These articles omit only one or fewer of the above-mentioned domains, or may look at these domains in a singular way, such as only asking about change in self-report rather than measuring a change compared to a baseline or identity process and identification types without measuring strength/magnitude.

Scoring was conducted by the first author during data extraction and charted alongside indicators for the presence of specific domains that were used in the explanatory count of each variable (e.g., number of articles examining ethnic identity or number of articles examining purchase behavior). When articles included results from multiple studies or surveys, the higher of the two scores was assigned. The scores and rationale were then reviewed by and discussed with the second author to ensure there was a consensus on scoring by article.

## 3. Results

### 3.1. Evidence Synthesis

**Figure 1:**
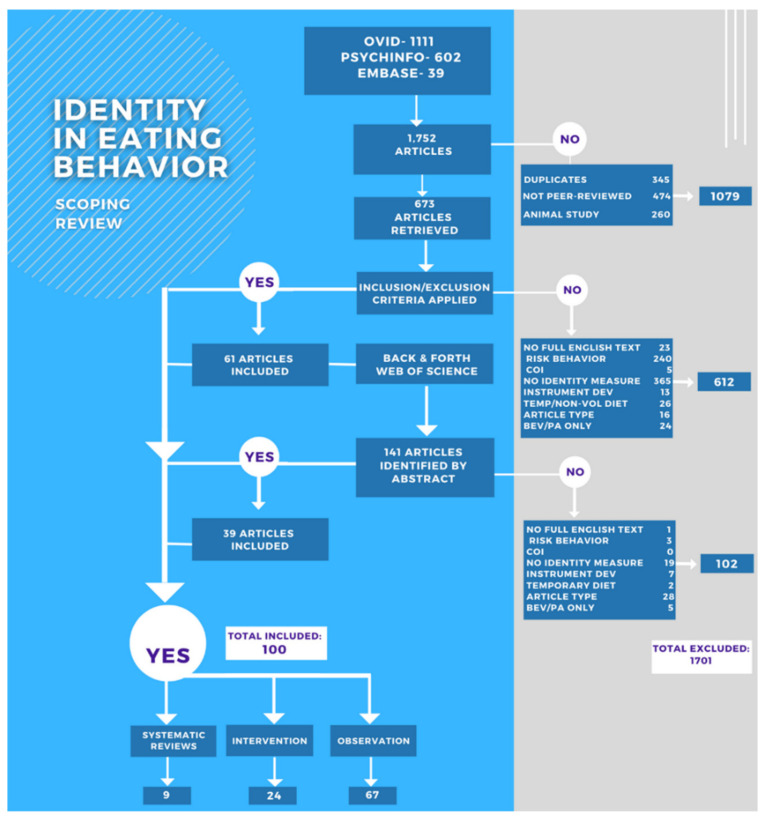
Flow chart of the exclusion and inclusion of articles in this scoping review.

**Figure 2:**
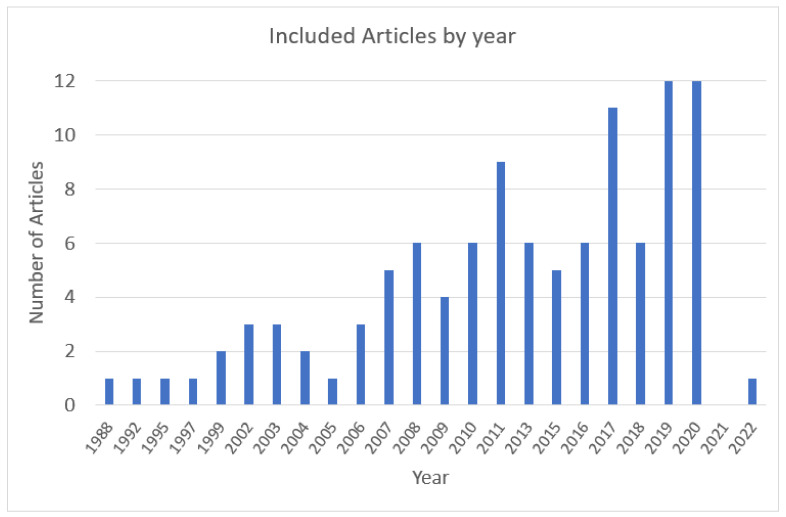
Range of years for studies in this review.

Details of the search results are included in Figure 1. Our search resulted in 1752 articles from the three primary databases. The initial review excluded 1079 articles (duplicates; dissertations, abstracts, and articles not published in peer-reviewed journals; and animal studies). After the inclusion and exclusion criteria were applied, an additional 294 articles were excluded, mainly due to them having no direct measurement of identity (166 articles). We conducted a back-and-forth search on the 61 articles using Web of Science. After reviewing abstracts for subjects relating to identity and eating behavior, 141 articles were retrieved. The application of the inclusion/exclusion criteria resulted in 39 additional papers, for a total of 100 papers included in this review (see flow chart, Figure 1). Most studies were published in 2007 or later (Figure 2).

### 3.2. Study Characteristics

The characteristics of the 100 articles included in this review are described in Table 1. A majority were observational studies and examined a single time point; 64% (*n* = 64) of the articles had a duration of a single occasion or were shorter than one month; 53% (*n* = 53) included predominantly female participants; and 27% (*n* = 27) studied students.

Many studies explored eating behaviors broadly as “healthy eating” (Table 1). At times, this was evaluated using food records and diet assessments [57,58,81,82] and quality indices and quality-scored food frequency assessments [41,83,84,85]. Other studies used fruit and vegetable intake as a proxy [36,49,51,53,54,57,58,59,60,81,86,87,88,89]. “Healthy eating behavior” (*n* = 33) overlaps with fruit and vegetable intake (*n* = 22) partially, but not entirely. Dieting for weight loss was also a frequently explored eating behavior (*n* = 26). A total of 19 studies focused on vegetarian diets or diets aiming at meat reduction, which were typically explored for their elective reduction for ethical, environmental, and prosocial reasons [39,47,48,61,62,63,64,65,90,91,92,93,94,95,96]. Cultural food consumption, often measured through the level of acculturation and strength of ethnic identity, was explored in 18 articles [35,37,63,65,66,84,97,98,99,100,101,102,103,104,105,106,139], but this was not exclusively assessed in articles focusing on ethnic identity (Table 2). Of the 16 articles on purchasing behavior, most examined the association between intent to purchase and self-identity categories, such as purchase of environmentally sustainable foods by “green consumers” or the purchase of culturally relevant foods as an indicator of the strength of ethnic identity [35,98,99,100,102,105,106,107,108,109,110]. In total, 12 articles considered shared meals as evidence of family or ethnic identity. Seven studies examined identity with respect to portion control. In contrast to prior reviews and meta-analyses [28,36,37,38,43,51], few studies examined identity and the consumption of individual nutrients, including sugar (*n* = 4), low/high levels of fat (*n* = 4), and energy (*n* = 4).

### 3.3. Operationalization of Identity

**Figure 3:**
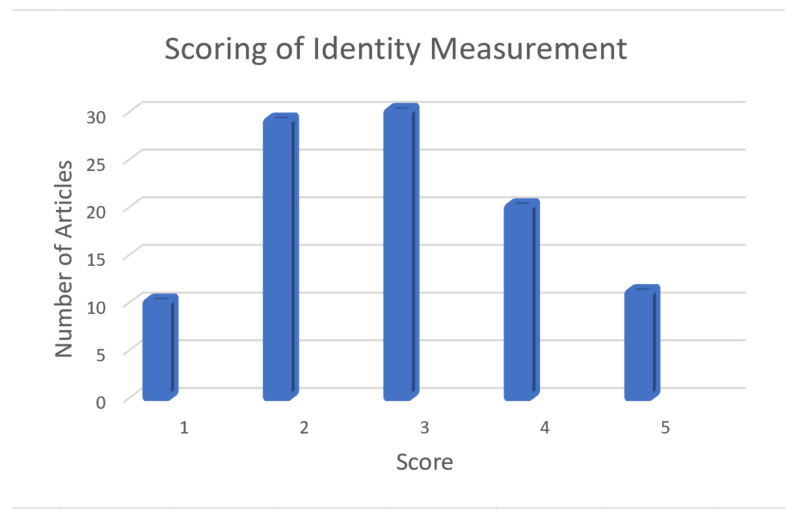
Distribution of scores related to the robustness of identity measurement in the articles included (*n* = 100).

The mean score of all articles was 2.9 on the scale from 1 to 5 based on the depth, complexity, and multi-dimensionality of the measures used. In total, 10 studies scored a “1” [6,36,37,66,67,68,69,104,111,112]; 29 scored a “2”, indicating the use of 1–2 basic questions about identity [42,51,52,53,54,60,70,71,72,84,85,87,88,96,97,100,102,103,113,114,115,116,117,118,119,120,121,122,139]; 30 received a score of “3” for their use of a common but short and non-complex identity instrument [29,33,36,39,40,47,49,62,73,74,75,76,81,82,91,93,98,99,101,110,123,124,125,126,127,128,129,130,131,132]; 20 received a “4” for a strong evaluation of identity and multiple types of identity, but lacked depth in their identity measure and/or comparison of identity effects to other constructs [32,38,41,43,44,59,61,63,64,65,79,83,86,89,90,94,95,106,133,134]; and 11 scored a “5” for a strong and in-depth measure of identity, including multiple types of identities [20,28,57,58,77,78,92,105,135,136,137] (Figure 3).

There was no consistent definition or type of identity among the articles reviewed (Table 2). Definitions of identity were predominantly derived from the foundational IT and/or SIT, with many studies directly introducing these theories [39,41,47,48,57,62,65,74,77,78,86,89,91,92,94,97,100,106,107,120,136]. The most common type of identity was one that was created for the behavior in question (*n* = 36), structured similarly to “I think of myself as a healthy eater”, “eating less junk food is important to me”, or “I am the type of person who watches what I eat”. These three questions are essentially identical to instruments used in the self-identity extension of TPB, and some studies explicitly stated their use of adaptations of this instrument [11,41,86]. While a variety of identity theories were used in the studies, the most common was TPB extended to include self-identity (*n* = 29). In some studies, the questions were adapted for a specific context, such as “ethnic self-identity” [66,97], or placed alongside questionnaires exploring other internal values such as pride [136], salience [20,39,46,60,65,78,122], centrality [39,48,57], or the interaction of multiple identities [39,43,132,135]. In 6 out of the 29 studies conducted using TPB, identity constructs came directly from one or both of IT/SIT [57,62,94,114,120,136]. The terms self-concept (*n* = 13) and self-schema (*n* = 8) were used in some studies, and were mostly or entirely the same as self-identity (Table 2).

Social identity was typically explored through degree of affinity with, and comparison to, the social group membership of the subject. This included the consideration of the role of that subject within their social group and/or an evaluation of their degree of affinity with, and compliance to, the norms and expectations of the group (Table 2). In total, 11 out of 31 studies on social identity did not examine group membership specifically, with most of those focused on how subjects appeal to “others” [20,25,37,73], how they conform to norms [65,78,95,105], a subject’s beliefs about their social role [36,60,135], and the desire to assimilate post immigration [70]. Not all studies examined group membership using a social identity approach [20,25,36,37,39,42,43,47,48,65,70,73,74,78,90,91,92,94,95,100,101,102,105,126,128,132,135,136]. Of those that did not, more than half focused on ethnic identity or the context of acculturation, most typically including notions of multiple identities and/or shifting identities from a sociological or anthropological perspective [35,40,63,66,97,99,103,115,139]. The others focused on voluntary behavior changes, such as school or workplace changes that were motivated by environmental sustainability [33,61,62,96,110], the construction of a vegetarian identity [64,93,133], or shifts in the self related to body image [52,67,72,89,122]. Similarly, one systematic review focused entirely on the role of identity shifts in eating behavior change [38]. Of note, of the ten studies in this review that explored multiple identities, nine examined social identity [39,43,47,48,74,90,102,132,135].

Ethnic identity was explored in twenty studies that examined the influence on behavior attributed to ethnic, cultural, or religious group affiliation (Table 2). These studies typically explored factors such as norms and conformity, traditions, the process of acculturation, and the drive to assimilate. Eleven studies looked at eating behaviors among immigrant populations within a new host culture [70,74,84,98,99,101,102,107,122,130,139]; five explored the specific intersection of cultural gender roles and eating behaviors [74,98,101,102,103]; four examined eating behaviors as a key distinguishing feature both between and within cultures [35,74,102,139]; four explored the role of ethnic identity in the eating behaviors of the children of immigrant communities [35,66,99,102]; and three looked at religious identity, specifically Muslim or Islamic identity [105,106,122]. Family identity (*n* = 4) was conceptually related to ethnic identity in all studies; however, none of the family identity papers included a direct measure or investigation of the strength of ethnic identity [40,104,115,134]. Additionally, for all four studies, the behavior evaluated was shared family meals and the transfer of cultural knowledge and traditions, and only one examined this from the lens of preserving a national identity [40].

Gender identity articles (*n* = 8) often explored group membership [37,51,74,90,95,103], well-known cultural norms such as the “thin ideal” and the muscular physique [51,90,118,125], and gender differences in cooking skill or behavior [51,103,118]. All studies explored the perceived masculinity or femininity of specific food choices, such as meat eating [90,95] or focused on choices such as the perceived gender-based attractiveness of the portion sizes of food eaten [37]. At least one study excluded respondents with non-binary gender self-identification [90].

Ethical identity, or identify based on personal values and ethics, was examined in nine studies (Table 2). Four focused on eating choices relevant to environmental sustainability [61,110,119,127], four on vegetarian or meat-reducing diet patterns [48,64,91,95], and two on food ethics as a politically and socially divisive factor [42,64]. While these studies were not directly focused on vegetarian diets or identity, all but one included it in their measurement or definition of ethical identity related to eating—the only study to focus on organic food purchasing [127].

Nearly half of the studies considered identity as changing (*n* = 42), such as level of assimilation to a host culture post-migration or in commitment to a newly adopted identity, while the majority (*n* = 58) treated identity as fixed (Table 2). While only ten articles included the measurement or analysis of concurrent or interacting identities (Table 2), the possibility of multi-fold identities was presented in the background or discussion of seven additional articles [51,54,76,100,101,106,115].

### 3.4. The Role of Identity in Eating Behaviors

In all studies evaluating the direct relationship between eating behaviors and identity, a significant relationship was observed. These relationships included the influence of strength of identity, identity salience, identity formation, reciprocal reinforcement between eating behaviors and identity, identity change, healthy diet adoption, intent to change or maintain eating behaviors, and adherence to dietary changes over time. In fact, only one study failed to find a significant effect for identity and eating behaviors, when analyzed as a mediator of social norms on eating behaviors [59]; however, the authors indicate that they believe their sample size was too low to adequately assess this mechanism. In subsequent studies conducted by the same group, strength of identity and identity centrality, especially when salience was manipulated, was associated with changes in eating intentions, food choice, and reported affiliations with the norm referent group [57]. Identity predicted individual sensitivity to social and subjective norms [20,53,57,78,85,92,93,105,110,126,128,132,134] and to medical advice related to eating behaviors [51,91,97,111,113], as well as resilience to adverse life events or routine interruptions [25,40,44,48,79,102]. Furthermore, in 15 studies, interventions to increase identity strength and salience improved self-concept, self-efficacy, perceived power, and adherence to short-term dietary changes that yielded improved clinical outcomes [25,28,67,68,71,72,79,80,86,87,97,101,119,126,132].

Thirty-two articles explored identity to help explain internal commitments to and maintenance of eating behaviors [6,25,28,29,33,37,38,39,46,49,53,63,67,68,75,85,86,89,90,91,93,95,97,100,105,111,113,126,130,131,137,139], both for factors underpinning the maintenance of healthy diet behavior and for intention to maintain a recent change, a high number given the relatively small number of articles with longer-term study durations (Table 1). One study found that a higher degree of social identity motivation (motivations related to social identity such as conformity or social norms) was associated with both a lower strictness to diet adherence and a tendency to over-report adherence [91]. Expanding on this, other articles discovered that stronger social identity was associated with adherence, except when the social influences are themselves the barrier, such as in the case of peer pressure or deviation from group norms [96,113]. Three other studies discussed the relationship between identity-based affinity with a health-care provider and compliance with, and trust in, medical advice (specifically ethnic identity [97,139] and gender identity [51]). These studies found that the centrality of identity and identity-relevant beliefs mitigated the adoption of and compliance with medical advice, and in some cases subject behavior was positively influenced by having medical providers that appeared to be group members or otherwise aligned with the subject’s identity [51,97,139].

Voluntary identity adoption and constructed identities were seen in studies on ethical identities. These studies demonstrated an individual’s ability to willfully create an identity and then engage in the identity processes that result in the centrality of that identity, reinforce that identity, and socially situate that identity including the behavior(s) that reciprocally express and reinforce it. For example, in studies on the “green consumer identity” [29,33,127,140] and the vegetarian identity [47,48,90], identities were mutually formed by values and by performing or eliminating behaviors—in many cases with identities defined by the specific avoidance or selection of products, behaviors, and self-labels [39,48,101,118,133]. These elective identities included substantial social identity signaling, with strong awareness of the significance of specific behaviors to group membership allegiance [47,48,49,90,91,92,133]. As such, these elective identities were observed to reinforce core internal values and were highly predictive of behavior [32,42,47,48,72,77,91,92,97,101,107,113,126,132,137]. These included ethical and/or vegetarian identities and the “Self-As-Doer” approach to identity, which all include the voluntary manufacture of identity and the positive reinforcement of relevant eating behaviors associated with that identity. Some studies examined complex self-identities that interrelated social and self-identity, such as in the Unified Model of Vegetarian Identity [47,48,49,90,91,92,133,141]. In these constructed identities, greater adherence and centrality were associated with stronger identification. Additionally, two studies included voluntary identities in the form of identification with the college in which they were enrolled [57,59].

Articles on assimilation, migration, and acculturation aimed to understand the factors supporting the maintenance of ethnic identity versus factors leading to conformity within the new host culture [70,74,84,98,99,104,122,139]. These studies demonstrate the complex influence of identity threat on behavior: in one case, the subjects felt more threatened by a loss of their original identity and were observed to eat more masculine identified foods and drink more alcohol to demonstrate and validate their identity [98]; in the other, the subjects responded to pressure to be seen as fully integrated into their new host culture, and thus in the experimental context were more likely to choose foods viewed as American, such as cheeseburgers, fries, and sodas [70]. This demonstrates the IT/SIT concept of identity salience—that overall composite identity results from the relative magnitude of a person’s priorities, values, and affiliations.

In several studies, salience of identity was seen to shift both naturally over time, and at other times to respond to cues [17,18,39,41,48,61,126], such as environmental contexts such as workplaces [61], and in response to researcher-manipulated identity challenges that examined whether participants would choose healthy-eating identity affirming or clashing foods, relative to the assessed strength of identity [17,18,39,41,48,57,65,78,126].

Many of the articles treated identity as the source of motivation to change eating behaviors, wherein motivation was derived from the pervasive internal need for identity-congruence [25,38,41,77,116]. Some studies described the formation or fortification of identity as a second process, alongside motivation, that helped make the change in eating behavior more relevant and central, thereby transforming motivation into behavioral action [28,57,69,87,93,96]. Some articles treated identity performance as a goal unto itself [25,29,75,77,87,127,140], whereas others treated identity as predictive of defining and pursuing goals and of being resilient to obstacles, routine interruptions, and major life events [6,35,40,44,48,60,77,82,89,123]. One article discussed goals as identities unto themselves, similarly to the constructed identities [116].

Identity was often explored in relation to the well-known constructs of social and peer norms and identity threats (including identity congruence challenges, attractiveness, and stigma). The relationship between social or group identity and norms and peer influences was examined in 29 studies [28,32,35,37,42,43,48,51,57,59,74,78,84,85,87,91,92,97,101,104,105,107,108,109,118,125,126,128,132]. Many of these studies considered eating behavior part of the expression of identity, such that behavioral deviations might indicate identity threats (*n* = 45) [6,32,35,37,38,40,42,43,46,47,48,51,52,53,58,61,64,66,69,70,72,73,89,90,91,92,93,94,96,97,98,99,100,102,103,104,106,113,118,119,121,125,131,132,137] or specific social group membership (*n* = 47) [33,35,38,39,40,42,43,47,48,51,53,57,58,59,61,62,65,66,67,72,74,78,84,89,90,91,92,93,94,95,97,99,100,101,102,103,105,106,110,115,126,128,132,133,134,136,139].

## 4. Discussion

Overall, the literature explores many different types of identity and includes wide variation in methods of defining, evaluating, and analyzing identity and its relationship with eating behaviors and change maintenance. In all but one of the articles reviewed, identity was strongly associated with, or helped to explain, eating behaviors or complementary internal processes that inform eating behaviors. Together, the findings from the work reviewed suggest that social and self-identities, as well as ethnic, ethical, eater-type, and other behavior-based identities, are associated with eating behavior change and maintenance. Failing to include identity may impact associations between other factors and eating behaviors; additionally, opportunities to create identity-based interventions may be missed. These findings also confirm that with respect to eating behaviors, identities are multi-fold, and identity, as well as identity salience, may shift in an individual over time and may be particularly responsive to intervention.

### 4.1. Operationalization of Identity

Identity was most often explored simplistically, and the small number of more recent studies accounted for the influence of multi-fold identities that may conflict, shape, or augment identity performance. These studies aimed to address multiple aspects of identity by developing methods with which to cross-compare different internal levers and to understand identity formation, change, and multiplicity. Studies with less comprehensive measures of identity tended to regard identity as a type of motivation or a characteristic of behavior and may confuse identity with other behavioral constructs, such as cultural norms, self-esteem, and habits. Consistent with the previous reviews on identity and eating behaviors, there was no consistent application of identity type, nor were consistent methods used for evaluating identity, the treatment of identity as changeable or fixed, or the evaluation of the process of forming or changing identities and related behaviors. As such, fewer papers followed changes in identity or behavior during the lifespan of the subjects. The lack of inclusion of these components and identity measures as a whole may exaggerate confounding from these variables in the manufactured environment of the research setting. There is a need to increase the dimensionality and standardize the evaluation of identity in order to fully understand its explanatory power for eating behaviors and healthy eating behavior changes. Standardized and complex measures of identity would also help ensure a fuller understanding of how identity processes can be used to assist in the adoption of new eating behaviors and adherence to behavior changes.

Identity theories will likely be useful in improving operationalization. Most studies were informed by IT or SIT; however, only some investigated multiple identities [126,142] or how salience and priorities shift depending on context, life changes, self-determined goals, and other factors [15,17,21,40,48,77,86].

Self-identity is one of the most common extensions of the TPB and has been shown in multiple meta-analyses to independently explain an additional 4–6% of the variance in intention and up to 9% when controlling for past behavior [28,30,31,32,33,34]. Most of the studies reviewed relied on a modified version of the extension of TPB, itself the most commonly used theoretical approach to identity, and therefore most studies assumed that identity was an a priori construct of the self [29,83,126,135,136]. However, one meta-analysis discussed how its findings suggest a need to disentangle identity from the pre-formed aspects of behavior in TPB such as attitude and intention [28]. In a study based on TPB that had more comprehensive identity measurements, identity explained up to 28.5% of the variance in intention and 5.7% of the variance in the behavior itself [83]. This is meaningful, given that, through meta-analytic methods, TPB only explained 21.2% of variance in eating behaviors and 52.4% of intention [34,87]. Theory advancement papers call for the examination of complex, shifting, and evolving identities [25,35,44,45,46,47,48,49], and this review confirms the need for additional research in this area.

It will also be useful in future research to understand how identity may be best incorporated into other traditional health behavior change theories besides TPB. In this review, we found few studies that included Social Cognitive Theory, Self-Categorization Theory, or the Transtheoretical Model. There is also a need for additional longitudinal and longer-term studies, as well as those that include real-time behavior adoption, performance, and maintenance over time. Another area that merits further research is illness identity. In the few studies that examined this, identity measurements were constructed around behaviors aimed at managing illness, such as checking blood sugar [113,117], and not around internal factors as indicated by the psychological theories of identity.

The use of identity and the theoretical applications of identity in research on eating behaviors does not represent the full scope of identity-relevant theory as applied in disciplines such as health psychology. For example, none of the included studies investigated formative identity development constructs, and in general the formation of identity was primarily assumed a priori or was examined as a volitional eater-type identity. This is a notable absence in the literature on eating behaviors in children. It also represents a fairly distinct difference in how identity is defined and studied more broadly compared to how it has been examined in the literature on eating behaviors. Additional research is therefore necessary in order to understand how the full range of identity-relevant theories may apply to eating behaviors.

### 4.2. Role of Identity in Eating Behaviors

Out of thousands of articles evaluating eating behaviors, only the 100 articles included in this review aimed to understand the role of identity in eating behaviors. Additionally, the articles within this review had considerable overlap in the authors’ publishing on these topics, which suggests that this approach is not yet widely practiced.

We regarded identity measurements as incomplete if they considered only one point in time or place or only one sub-component of identity and if they did not consider measurements of either strength, internal processes such as salience or formation, or subjective importance. Some studies included many or most of these components, while the majority attempted to use a single component of identity to describe a larger and more complex construct. While these components have demonstrated associational and explanatory value when considered independently, our findings suggest the importance of considering more robust and complete measures in order to better understand the role of identity in eating behaviors. In particular, we found that eating behavior and identity have reciprocal and mutually reinforcing roles, and as such omitting the more complex facets of identity may inaccurately attribute directionality to these specific sub-components, including during experimental manipulations. Further research should investigate identity formation and change in order to better understand this reciprocal relationship. Our findings also suggest that future research might incorporate a wider theoretical perspective to allow for an understanding of more complex relationships between eating behavior and identity.

There is growing interest in understanding eating behaviors centered around personal values [29,48,138,143,144,145]. Understanding the effect of these internal values on long-term eating behaviors and the ways they shift over time will shed light on the reciprocal relationship between internal drivers (such as personal priorities, motivation, and goals) and behavioral performance and ultimately on outcomes such as health, quality of life, self-identity, and social identity. Obtaining a greater understanding of the internal drivers and moderators of eating behavior has practical applications in research and clinical practice.

### 4.3. Limitations

This review consolidates and synthesizes extant knowledge across disciplines to translate and streamline the effective application of identity-sensitive approaches in multiple sectors. As is the case with much research, identity still needs to be studied among more diverse populations. Many of the included studies used convenience samples, such as college students, or samples from populations that were largely female, white, and affluent. Given the clear importance of identity to communities with higher identity salience, those who routinely experience identity-based discrimination or threat, and those who live in conditions where identity may be a central component of daily life, there is a great need to consider identity-sensitive approaches to health behavior in research and practice. Another significant limitation of this review is that in an effort to blend study types, methodological approaches, and disciplines for a comprehensive landscape of identity in eating behaviors, we were not able to mathematically analyze the effect of identity with a meta-analysis. Finally, identity is used to examine many different eating behaviors; however, “healthy eating” behavior was typically undefined. While some studies use fruit and vegetable intake as a proxy for “healthy eating”, others focus on specific interpretive aspects of what may comprise a healthy diet, such as low-fat food or “junk food” avoidance, representing opinions which may change over time as the evidence on healthy diet composition evolves. Therefore, this approach may be less relevant to understanding the relationship between identity and healthy eating where a more direct measure of nutritional literacy would suffice. However, the growing volume of constructed identity research presents an opportunity to explore how identity can help develop and change intrapersonal relationships to food and eating habits.

## 5. Conclusions

This review extends prior ones by including studies of a broader range of eating behaviors and more complex measures of identity. The findings corroborate previous reviews that found consistent, significant associations between identity and eating behaviors. The findings also suggest that while even limited identity measures yield substantive results, a more comprehensive consideration of identity would likely yield more accurate results and have even greater explanatory power. Future research is needed to establish more robust measures of identity. Intervention studies to establish the role of identity in promoting long-term changes in eating behaviors are also warranted.

## Figures and Tables

**Table 1:** Study characteristics (*n* = 100).

Characteristics ^a^		Number of Studies	References
Study type	Intervention	24	[57,58,59,60,61,62,63,64,65,66,67,68,69,70,71,72,73,74,75,76,77,78,79,80]
	Observational	67	[6,20,29,32,33,39,40,41,42,81,82,83,84,85,86,87,88,89,90,91,92,93,94,95,96,97,98,99,100,101,102,103,104,105,106,107,108,109,110,111,112,113,114,115,116,117,118,119,120,121,122,123,124,125,126,127,128,129,130,131,132,133,134,135,136,137,138]
	Systematic review	9	[28,36,37,38,43,51,52,53,54]
Study duration ^b^	One-time	46	[20,29,33,40,42,57,59,65,78,84,85,88,90,91,92,93,94,95,96,97,99,100,103,106,107,108,109,110,111,112,113,114,116,117,118,119,121,122,127,128,129,131,132,133,136,137]
	<1 month	18	[39,62,67,69,70,73,74,77,81,82,83,86,98,101,124,126,135,139]
	1–6 months	13	[32,41,60,61,64,68,71,75,76,80,87,120,123]
	>6–12 months	6	[6,66,79,89,102,130]
	>1 year	5	[63,72,104,115,125]
Population	All men ^c^	3	[51,79,98]
	>2/3 women	53	[6,33,40,41,42,54,57,58,59,62,63,64,67,68,69,71,72,73,74,75,76,77,78,80,81,83,86,87,88,89,91,95,97,99,101,102,103,104,110,112,115,119,122,123,124,126,127,128,129,131,133,137]
	Students	27	[32,41,57,58,59,60,62,64,69,70,74,75,77,78,81,83,86,87,96,110,116,123,124,126,129,132,135]
	Children/adolescents	8	[58,60,66,72,84,85,125,128]
	Adults (sex mixed; non-student)	31	[20,28,29,36,37,38,39,43,52,53,61,65,82,90,92,94,100,105,106,107,111,113,114,117,118,120,121,130,134,136,139]
Eating behaviors ^d^	Healthy eating	33	[37,38,41,43,51,52,53,54,58,60,63,70,74,75,76,77,81,82,83,84,85,86,87,89,111,112,116,117,122,124,126,132,135]
	Dieting for weight loss	26	[6,38,44,52,53,58,60,67,68,69,71,72,73,75,76,79,80,89,113,120,123,125,128,131,135,137]
	Vegetarian or meat reduction	19	[39,47,48,61,62,63,64,65,86,88,90,91,92,93,94,95,96,98,102]
	Cultural food choice	18	[35,63,65,66,70,74,84,97,98,99,101,102,103,105,106,107,130,139]
	Fruit and vegetable intake	22	[29,36,41,51,53,54,57,58,59,60,66,72,75,81,85,86,87,88,89,98,129,130]
	Food purchasing	16	[33,61,98,100,101,102,105,106,107,108,109,110,114,119,127,136]
	Shared meals	12	[20,35,40,47,85,99,100,102,103,104,115,134]
	Portion size	7	[53,91,99,108,109,115,139]
	Low-/high-fat diets	4	[32,75,121,124,136]
	Sugar intake	4	[53,72,75,124]
	Energy intake	4	[74,75,89,120]

^a^ Characteristics categories are not mutually exclusive and were derived inductively. ^b^ *n* = 91 because systematic reviews not included in count. ^c^ One study included 2 women, for 96% men only. Systematic reviews contained some sex-exclusive studies within their reviews; this is not reflected in this total. ^d^ Not mutually exclusive—many studies included more than one eating behavior.

**Table 2:** Operationalization of identity (*n* = 100).

Characteristic		Number of Studies	References
Identity type ^a^	Behavior-based identity	36	[6,28,29,38,41,52,53,61,62,70,72,73,75,76,77,81,82,83,86,87,88,89,90,92,93,94,96,111,112,116,117,131,132,133,135,137]
	Social identity	30	[20,36,37,39,42,43,54,57,59,60,65,70,73,74,78,89,90,91,92,94,95,100,101,102,108,126,128,132,135,136]
	Self-identity	24	[20,28,29,32,39,43,54,57,66,81,87,95,109,114,119,120,121,125,127,129,132,135,136,137]
	Ethnic/Cultural identity	20	[35,63,65,66,70,74,84,97,98,99,101,102,103,105,106,107,122,130,134,139]
	Self-concept	13	[38,54,58,67,68,69,71,77,79,80,81,85,124]
	Multiple identities	10	[39,43,47,48,74,77,90,102,132,135]
	Gender identity	8	[37,51,74,90,95,103,118,125]
	Self-schema	8	[35,44,111,113,116,123,124,135]
	Ethical identity	9	[42,48,61,64,91,95,110,119,127]
	Family identity	4	[40,104,115,134]
Identity theory ^a^	Theory of Planned Behavior with identity extension	29	[28,29,32,33,36,43,57,59,60,61,65,68,74,76,87,92,94,96, 103,104,111,112,114,120,123,124,125,132,133]
	Social Identity Theory	15	[39,57,65,73,74,77,78,89,91,92,94,97,100,106,107]
	Identity Theory	8	[39,41,62,77,86,100,120,136]
	Other identity theory ^b^	20	[20,25,35,38,42,44,45,46,49,61,68,71,72,75,79,89,90,102,117,133]
	Other theory, not specifically identity-related ^c^	14	[20,38,58,63,64,65,85,89,93,98,99,102,132,134]
Identity characteristics	Fixed	58	[6,29,32,33,36,37,39,40,41,43,51,53,54,59,63,65,72,73,78,80,81,82,83,84,85,86,87,88,90,94,96,97,100,103,105,106,107,108,109,110,111,112,113,114,115,116,118,119,121,122,123,124,126,127,128,129,132,136]
	Changing	42	[20,28,38,42,52,57,58,60,61,62,64,66,67,68,69,70,71,74,75,76,77,79,89,91,92,93,95,98,99,101,102,104,117,120,125,130,131,133,134,135,137,139]

^a^ Not mutually exclusive; many studies included more than one identity type. ^b^ ‘Other Identity Theory’ refers to theories of identity other than IT and SIT, none of which had more than 4 studies operationalizing approaches from these theories. These included 13 other theories: Unified Model of Vegetarian Identity; Social Cognitive Theory with identity extension; Self-Categorization; Self-As-Doer Theory; Situated Identity; Identity-Based Motivation Theory; Identity Process Theory; Moral Foundation Theory; Personal Construct Theory; Self-Congruity Theory; Self-Enhancement Theory; Spoiled Identity Theory; Identity-Based Motivation. ^c^ ‘Other Theory, not specifically identity related’ refers to behavioral theories that are not identity-specific or theories that do not include identity as a component. These included ten theories: Integrated Model of Behavior Change; Theory of Interpersonal Behavior; Theory of Normative Social Behavior; Deleuzian Body Model; Precede-Proceed; Grounded Theory; Cognitive Control Theory; Self-Categorization Theory; Consumer Culture Theory; Interactionism.

## Data Availability

Data may be available upon request from qualified research teams.

## Supplementary Materials

The following supporting information can be downloaded at: https://www.mdpi.com/article/10.3390/nu14173456/s1, Supplemental Material 1: Search Strategy for Ovid MEDLINE(R) <1946 to March 2022>; Supplemental Material 2: Preferred Reporting Items for Systematic reviews and Meta-Analyses extension for Scoping Reviews (PRISMA-ScR) Checklist.

## Abbreviations

DALY	disability-adjusted life years
IT	Identity Theory
SIT	Social Identity Theory
TPB	Theory of Planned Behavior
